# Cell-Free Spent Media Obtained from *Bifidobacterium bifidum* and *Bifidobacterium crudilactis* Grown in Media Supplemented with 3′-Sialyllactose Modulate Virulence Gene Expression in *Escherichia coli* O157:H7 and *Salmonella* Typhimurium

**DOI:** 10.3389/fmicb.2016.01460

**Published:** 2016-09-22

**Authors:** Pauline Bondue, Sébastien Crèvecoeur, François Brose, Georges Daube, Marie-Christine Seghaye, Mansel W. Griffiths, Gisèle LaPointe, Véronique Delcenserie

**Affiliations:** ^1^Department of Food Science, Fundamental and Applied Research for Animal and Health, Faculty of Veterinary Medicine, University of Liège Liège, Belgium; ^2^Department of Pediatrics, University Hospital Liège-Notre-Dame des Bruyères Belgium; ^3^Canadian Research Institute for Food Safety, University of Guelph Guelph, Canada

**Keywords:** *Bifidobacterium bifidum*, *Bifidobacterium crudilactis*, bovine milk oligosaccharide, *Escherichia coli* enterohemorragic O157:H7, *Salmonella enterica* serovar Typhimurium, virulence expression, 3′-sialyllactose, whey

## Abstract

Complex oligosaccharides from human milk (HMO) possess an antimicrobial activity and can promote the growth of bifidobacteria such as *Bifidobacterium bifidum* and *Bifidobacterium longum* subsp. *infantis*. In addition, fermentation of carbohydrates by bifidobacteria can result in the production of metabolites presenting an antivirulence effect on several pathogenic bacteria. Whey is rich in complex bovine milk oligosaccharides (BMO) structurally similar to HMO and *B. crudilactis*, a species of bovine origin, is able to metabolize some of those complex carbohydrates. This study focused on the ability of *B. bifidum* and *B. crudilactis* to grow in a culture medium supplemented in 3′-sialyllactose (3′SL) as the main source of carbon, a major BMO encountered in cow milk. Next, the effects of cell-free spent media (CFSM) were tested against virulence expression of *Escherichia coli* O157:H7 and *Salmonella enterica* serovar Typhimurium. Both strains were able to grow in presence of 3′SL, but *B. crudilactis* showed the best growth (7.92 ± 0.3 log cfu/ml) compared to *B. bifidum* (6.84 ± 0.9 log cfu/ml). Then, CFSM were tested for their effects on virulence gene expression by *ler* and *hilA* promoter activity of luminescent mutants of *E. coli* and *S*. Typhimurium, respectively, and on wild type strains of *E. coli* O157:H7 and *S*. Typhimurium using RT-qPCR. All CFSM resulted in significant under expression of the *ler* and *hilA* genes for the luminescent mutants and *ler* (ratios of −15.4 and −8.1 respectively) and *qseA* (ratios of −2.1 and −3.1) for the wild type strain of *E. coli* O157:H7. The 3′SL, a major BMO, combined with some bifidobacteria strains of bovine or human origin could therefore be an interesting synbiotic to maintain or restore the intestinal health of young children. These effects observed *in vitro* will be further investigated regarding the overall phenotype of pathogenic agents and the exact nature of the active molecules.

## Introduction

Due to the influence on the microbiota of carbohydrate source present in food, breast-fed children are generally in better health than children fed with formula (Arrieta et al., 2014; Smilowitz et al., 2014; Scott et al., 2015). Human milk oligosaccharides (HMO) are complex oligosaccharides found in human milk. Their concentration can reach 15 g/l and more than 500 structures have been identified (Pacheco et al., 2015). These HMO, produced in mammary glands, cannot be metabolized either by the host or most bacteria, while bifidobacteria species have enzymatic activity able to degrade specific α- and β-bonds (Garrido et al., 2013). These bacteria such as *Bifidobacterium bifidum* subsp. *infantis* or *Bifidobacterium bifidum* are mainly found in the feces of breast-fed children. Indeed, the fecal microbiota of breast-fed child contain more than 75% bifidobacteria (Di Gioia et al., 2014). In addition to facilitating the growth of beneficial bacteria such as bifidobacteria, HMO can bind with pathogenic virus or bacteria, limiting adhesion to intestinal epithelium and therefore decreasing pathogens colonization by promoting natural excretion (Smilowitz et al., 2014). Also, other metabolites produced by lactic acid bacteria and bifidobacteria are able to inhibit virulence gene expression of *Escherichia coli* O157:H7 (Medellin-Pena and Griffiths, 2009; Zeinhom et al., 2012), *Salmonella enterica* serovar Typhimurium SA 941256 (Medellin-Pena et al., 2007; Bayoumi and Griffiths, 2012; *S*. Typhimurium) and *Campylobacter jejuni* (Mundi et al., 2013).

Five monosaccharides can be found in different HMO structures: glucose (Glc), galactose (Gal), N-acetylglucosamine (GlcNAc), fucose (Fuc), and sialic acid, also called N-acetylneuraminic (NeuAc). High quantities of lacto-N-biose type I (LNB: Galβ1-3GlcNAc) and fucosylated HMO are an important characteristic of human milk (Chichlowski et al., 2011; Garrido et al., 2013; Dotz et al., 2014; Smilowitz et al., 2014). *B. infantis* is able to fully degrade HMO intracellularly, contrary to *B. longum, B. breve*, and *B. bifidum* (Sela and Mills, 2010; Underwood et al., 2015). It possesses genes encoding specific transporters and four enzymes necessary for HMO degradation (α-fucosidase, α-sialidase, β-galactosidase, and β-N-hexosaminidase) (Sela et al., 2008; Sela, 2011; Smilowitz et al., 2014). HMO degradation by *B. bifidum* occurs outside the cells. Indeed, *B. bifidum* possesses a lacto-N-biosidase, which cleaves LNB from HMO. LNB is internalized using a specific transport system and is then degraded using LNB-phosphorylase (Sela, 2011; Smilowitz et al., 2014). Therefore, *B. infantis* and *B. bifidum*, despite their different gene clusters, both grow very well in the presence of HMO as sole source of carbon (Asakuma et al., 2011; Barile and Rastall, 2013).

Common oligosaccharides used in infant formula are galacto-oligosaccharides (GOS) and fructo-oligosaccharides (FOS), including inulin. GOS are composed of Glc and Gal, while FOS are composed of fructose and Glc. Their structures are very simple and linear. They are also bifidogenic, but because of their simple structure, they can also be consumed by other members of the intestinal microbiota such as *Bacteroides* spp. or *Clostridium* spp. (Chichlowski et al., 2011; Scholtens, 2014). This is probably why the fecal microbiota of formula-fed children contains only 30% bifidobacteria (Di Gioia et al., 2014) and the species that are present are different from those observed in the feces of breast-fed children. The predominant bifidobacteria in formula-fed children are those encountered in adult feces, such as *Bifidobacterium longum* subsp. *longum* and *Bifidobacterium adolescentis*, which present a less diverse enzymatic arsenal (De Vuyst et al., 2013).

Bovine milk oligosaccharides (BMO) can have similar composition and branching as HMO, so they could share some common properties. In addition, *B. infantis* or *B. bifidum* can grow in the presence of these carbohydrates (Sela, 2011; Milani et al., 2014). More than 60 BMO have been identified so far (Pacheco et al., 2015) and whey, a by-product of the dairy industry, is an important low cost source of BMO (Barile et al., 2009; Zivkovic and Barile, 2011). Furthermore, *Lactobacillus acidophilus* La-5 grown in medium supplemented with dairy ingredients such as whey, presented a protective effect in EHEC-infected mice (Zeinhom et al., 2012). However, BMO concentration in bovine milk is lower than HMO concentration in human milk (Barile et al., 2009; Tao et al., 2009; Kelly et al., 2013). Also, fucosylation occurs at very low frequency compared to sialylation, which is contrary to human milk (Tao et al., 2008). Even if the degree of polymerization in BMO is lower than in HMO, they are also protected by α- and β-bonds which are less accessible to other bacteria (Chichlowski et al., 2011). One of the most important BMO found in cow milk is 3′-sialyllactose (3′SL: NeuAcα2-3Galβ1-4Glc) and its concentration in colostrum can reach 0.85 mg/ml (Nakamura et al., 2003; Urashima et al., 2013). The majority of bifidobacteria grows only in anaerobic conditions, an environment very difficult to reproduce on an industrial scale. In addition, they have to survive the acidity of the stomach, bile salts and pancreatic enzymatic activity of the digestive tract in order to reach the colon where they will consume oligosaccharides. *Bifidobacterium crudilactis* FR/62/B/3, a species isolated from raw cow's milk cheese, is oxygen tolerant (Daube et al., 2006; Delcenserie et al., 2013). The genome encodes enzymes degrading BMO (Delcenserie et al., 2007; Milani et al., 2014). These strains from bovine milk could be an interesting source of probiotics for formula supplementation (Delcenserie et al., 2013).

The type III secretion system (T3SS) has a major role in virulence expression of *S*. Typhimurium and *E. coli* O157:H7 by injecting effector proteins in intestinal cells and forming attaching and effacing (AE) lesions on host enterocytes (Bayoumi and Griffiths, 2012). The proteins produced by *S*. Typhimurium are controlled and activated by *hilA* and *sopD* genes and the *ssrB2* gene is a major regulator of the T3SS (Guri et al., 2016). Regarding *E. coli* O157:H7, AE lesions are controlled by a pathogenicity island named locus of enterocyte effacement (LEE) through *ler* gene, which is regulated by *qseA* gene also implicated in quorum sensing (Medellin-Pena et al., 2007). The gene *luxS*, major regulator of quorum sensing and influencing the *qseA* and *ler* genes, is also involved in expression of genes encoding flagella and biofilm formation (*fliC*) or synthesis of shiga-toxin (*stxB2*) (Kaper et al., 2004; Wood et al., 2006).

The aims of this work were therefore to study the growth potential of *B. crudilactis* FR/62/B/3 compared to *B. bifidum* BBA1 in culture media supplemented with whey or 3′SL and to evaluate the effects of CFSM on virulence expression of *Escherichia coli* O157:H7 and *Salmonella* Typhimurium. Because of its bovine origin, the hypothesis is that *B. crudilactis* FR/62/B/3 can metabolize components present in whey, especially BMO, as explained previously. *B. bifidum* BBA1, a strain isolated from breastfed children feces, was chosen to provide a comparison with a strain of human origin as this strain should be able to use BMO or 3′SL as a source of carbon, due to their similarity with HMO (Zivkovic and Barile, 2011). Tanimomo et al. (2016) developed a culture medium answering to the specificities of *B. crudilactis* FR/62/B/3. This formula, more suited to bifidobacteria from bovine origin, was used in this study as an optimized medium in which different sources of carbohydrates have been tested. The effects of CFSM obtained from theses cultures on intestinal pathogens virulence were firstly investigated using rapid-testing on a luminescent reporter mutants. Next, virulence gene expression was more deeply investigated on several virulence genes using RT-qPCR on wild pathogenic strains. A special attention was given to the controls (unfermented media). Indeed, some nutrients have previously been shown to have a repressive effect on virulence gene expression of *E. coli* O157:H7 (Delcenserie et al., 2012) and therefore, it was important to exclude that potential effect from the observed results with the fermented media.

## Materials and methods

### Bacterial strains and growth conditions

*Bifidobacterium bifidum* BBA1 was isolated from feces from a breast-fed child (CHU - Hôpital des Bruyères, Liège, Belgium) and *B. crudilactis* FR/62/B/3 from Saint-Marcellin, a raw cow milk cheese from Vercors (France). Both strains were stored at −80°C and grown on De Man, Rogosa, and Sharpe (MRS) medium (Oxoid, Hampshire, UK) supplemented with cysteine-HCl (0.5 g/l) and mupirocin (0.08 g/l) at 37°C for 48 h in an anaerobic workstation (Led Techno, Heusden-Zolder, Belgium) containing 10% H_2_, 10% CO_2_, and 80% N_2_. Several successive cultures, in the same conditions as described previously, have been realized in MRS broth, prior to use. Pathogenic enterohaemorrhagic *E. coli* (EHEC) strain O157:H7 ATCC 35150 (${\mathrm{stx}}_{2}^{+}$) and *S. enterica* serovar Typhimurium strain ATCC 14028 were stored at −80°C and grown in Luria Bertani (LB) media (Sigma-Aldrich, Diegem, Belgium). Two reporter mutants, *E. coli* O157:H7 ATCC 43888 (*stx*^−^, LEE:*lux*) containing plasmid LEE1-luxCDABE and resistant to ampicillin (Amp^r^) and kanamycin (Kan^r^) and *S*. Typhimurium SA 941 256 containing plasmid pSB377 (*hil*A::*lux*CDABE; Amp^r^) were designed by Medellin-Pena et al. (2007) and Bayoumi and Griffiths (2010), respectively. Both strains were from the Canadian Research Institute for Food Safety Collection and were grown under aerobic conditions at 37°C in brain heart infusion (BHI) broth (Bio-Rad, Marnes-la-coquette, France) supplemented with ampicillin (50 mg/l). A medium optimized for *B. crudilactis* FR/62/B/3, called MRS2 (Tanimomo et al., 2016) was considered as the reference medium for this study (Table 1) and was modified by removing or replacing glucose: MRS2 without any glucose (MRS2 G) (control), MRS2 with a mix of glucose and whey (MRS2-Wh) and MRS2 with 3′SL (MRS2-3′SL) as the only source of carbohydrate (Table 1). Whey was collected at the beginning of a curdling process of a Belgian cheese factory (Liège area, Belgium). The quantity of lactose in MRS2-Wh medium was estimated to 25 g/l, based on lactose concentration of sweet whey (50 g/l of lactose; Food and Agriculture Organization/Organisation Mondiale de la Santé, 1998). However, mature bovine milk contains only traces of BMO (Kelly et al., 2013). The 3′SL, added to MRS2-3′SL, was provided by Carbosynth laboratory (Berkshire, UK). The concentration of 0.85 g/l was chosen to be close to natural concentrations found in colostrum (Nakamura et al., 2003). *B. bifidum* BBA1 and *B. crudilactis* FR/62/B/3 were grown in three independent experiments under the same anaerobic conditions as previously at 37°C for 48 h. Five log/ml of bifidobacteria from a fresh 48 h culture of bifidobacteria were inoculated into the fresh media (1% v/v). The concentration of 5 log/ml was confirmed by plating several dilutions of bifidobacteria at day 0 post inoculation. Bacterial growth was determined by viable counts after 48 h incubation. Cell free spent media (CFSM) were obtained after two centrifugation steps at 5000 × (Eppendorf Centrifuge 5804, Hamburg, Germany) for 10 min. Supernatants were then sterilized by filtration (Minisart® 0.45 μm and 0.2 μm, Sartorius, Vilvoorde, Belgium). Next, CFSM were freeze-dried (Virtis Benchtop 3.3 EL, SP Scientific, Suffolk, United-Kingdom) and rehydrated with sterile distilled water to obtain a 10x concentration. The same treatment was applied to non-fermented culture media (controls). The pH of rehydrated CFSM was adjusted to 7 using 1 M NaOH.

**Table 1:** **Composition of modified MRS2 media**.

	**MRS2 G**	**MRS2**	**MRS2-Wh**	**MRS2-3′SL**
Yeast extract (g/l)	15.5	15.5	15.5	15.5
Peptone of casein (g/l)	15.5	15.5	15.5	15.5
K_2_HPO_4_ (g/l)	0.9	0.9	0.9	0.9
KH_2_PO_4_ (g/l)	0.9	0.9	0.9	0.9
NaCl (g/l)	0.009	0.009	0.009	0.009
MnSO_4_.H_2_O (g/l)	0.17	0.17	0.17	0.17
MgSO_4_.7H_2_O (g/l)	0.007	0.007	0.007	0.007
FeSO_4_.7H_2_O (g/)	0.009	0.009	0.009	0.009
Tween 80 (ml/l)	0.9	0.9	0.9	0.9
Cysteine (g/l)	0.4	0.4	0.4	0.4
Glucose (g/l)	–	20	10	–
Whey (ml/l)	–	–	500	–
3′-sialyllactose (g/l)	–	–	–	0.85

### Measurement of LEE and *hilA* promoter activity

Both *E. coli* reporter strains were grown overnight in BHI broth supplemented with ampicillin (BHI-Amp). Each overnight culture was diluted 1:100 with fresh BHI-Amp broth supplemented (test samples) or not (control samples) with 10% concentrated CFSM obtained from fermented MRS2-3′SL. Two hundred microliters of each sample were distributed into triplicate wells of a sterile, opaque 96-well microliter plate (Corning 3610, Fisher Scientific, Ottawa, Ontario, Canada) and incubated at 30°C. Luminescence was measured every hour for 24 h, with a Victor multilabel counter (Wallac, PerkinElmer Life Sciences Canada, Woodbridge, Ontario, Canada). Luminescence was expressed in counts per second. Optical density (OD) was determined using a Genesys 20 spectrophotometer (Thermo scientific, Erembodegen, Belgium) adjusted to 600 nm.

### Contact between concentrated CFSM and wild type pathogenic strains

*E. coli* O157:H7 ATCC 43890 was grown in LB agar and a single colony was taken from the plate and incubated overnight in LB broth at 37°C with aeration. The same procedure was applied for *S*. Typhimurium ATCC 14028 using BHI medium. The cultures were homogenized and 50 μl were diluted in 4.5 ml of LB broth for *E. coli* and BHI broth for *S*. Typhimurium. Then, 450 μl of each concentrated CFSM was added to the bacterial suspensions. Triplicate cultures were incubated at 37°C for 4 h on a shaker at 150 rpm. Bacterial growth was determined by OD measurement at 600 nm. *E. coli* O157:H7 and *S*. Typhimurium were grown in LB and BHI broth alone, respectively, as controls.

### Gene expression analysis by RT-qPCR

The method was adapted with some modifications from Tellez et al. (2012), Mith et al. (2014) and Guri et al. (2016). After 4 h of incubation (Delcenserie et al., 2012), cells were collected by centrifugation at 5000 × g for 10 min at room temperature (Eppendorf Centrifuge 5804, Hamburg, Germany). The pellet was suspended in 100 μl TE buffer (10 mM Tris and 1 mM EDTA) containing 1% lysozyme (Roche, Mannheim, Germany). Samples were stored at −20°C overnight. On the next day, RNA was extracted using the RNeasy® Mini Kit (Qiagen, Antwerp, Belgium). DNA contamination was eliminated from each sample using the DNase I Recombinant RNase-free Kit (Roche Diagnostics GmbH, Mannheim, Germany). To inactivate the DNase, samples were heated at 75°C for 10 min. The quantity of RNA was determined by measuring the absorbance at 260 nm using a Nanodrop 2000 Spectrophotometer (Thermo Scientific, USA). The purity and quality of RNA were verified by measuring the ratio of absorbance (260 nm/280 nm) and by using agarose gel electrophoresis (Eurogentec, Seraing, Belgium). The concentration of RNA used for reverse transcription was normalized to 100 ng/μl for *E. coli* and to 50 ng/μl for *S*. Typhimurium. Next, the RNA was subjected to reverse transcription polymerase chain reaction (RT-PCR) using a high-capacity cDNA Reverse Transcription Kit (Applied Biosystems, Ghent, Belgium). Briefly, 1 μg of RNA was reverse transcribed with 0.8 μl of desoxyribonucleoside triphosphate (dNTP; 100 mM), 1 μl of Multiscribe® Reverse Transcriptase (50 U/μl), 2 μl of 10X RT Random Primers and 2 μl of 10X RT Buffer in an adjusted total volume of 20 μl. For each sample, a no-RT control was included to confirm the absence of DNA contamination. Synthesis of cDNA was performed in a Mastercycler Gradient Thermocycler (Flexigene, Cambridge, UK) under the following conditions: 25°C for 10 min, 37°C for 120 min, 85°C for 5 min and a cooling step at 4°C. Then, cDNA was stored at −20°C.

To study the effects of bioactive molecules present in culture supernatant on gene expression of *E. coli* O157:H7 ATCC 43890, the expression of genes *ler* (involved in attaching effacing lesions), *fliC* (involved in mobility), *stxB2* (encoding subunit B of Shiga-toxin 2), *luxS* (major regulator of quorum sensing and producing AI-2), and *qseA* (involved in quorum sensing and regulator of LEE expression) was determined using qPCR. The same method was used to study effects on *S*. Typhimurium virulence gene expression of *hilA* (invasion protein regulator), *ssrB2* (Type III secretion system regulator), and *sopD* (secreted effector protein). Quantitative PCR amplification was conducted using the GoTaq® qPCR Master Mix (Promega, Leiden, Netherlands) and using the ABI 7300 Real Time PCR System (Applied Biosystems, Singapore) for *E. coli* or the Light Cycler 480 (Roche Diagnostics, Mannheim, Germany) for *S*. Typhimurium. The primers were synthesized by Eurogentec (Liège, Belgium) and have been used in previous studies (Table 2). The RT-qPCR was performed in a total volume of 20 μl, containing 10 μl of GoTaq® Master Mix, 5.75 μl of molecular grade water, 1 μl of forward primer (10 μM), 1 μl of reverse primer (10 μM), 0.25 μl of carboxy-X-rhodamine (30 μM), and 2 μl of diluted cDNA. The qPCR conditions for *E. coli* were: initial denaturation at 95°C for 3 min; denaturation, annealing and elongation repeated 45 times: 95°C for 15 s, 58°C for 30 s and 72°C for 45 s; melting curve program: 60–95°C with a heating rate of 0.1°C/s. The qPCR conditions for *S*. Typhimurium were: denaturation at 95°C for 10 min; 40 cycles of amplification and quantification: 95°C for 30 s, 56°C for 30 s and 72°C for 30 s; melting curve program: 60–95°C with a heating rate of 0.1°C s. The annealing temperature, optimized at 56°C, was determined experimentally. Each specific amplicon was validated for the presence of a single melting temperature peak and a single band of expected size on a 2% agarose gel after electrophoresis. Cycle threshold (C_t_) values were determined using the ABI 7300 System SDS Software for *E. coli* and the Light Cycler Software 480 version 1.5 for *S*. Typhimurium. Four housekeeping genes were tested for *E. coli*: *gnd* (6-phosphogluconate deshydrogenase), *gst* (glutathione S-transferase), 16S gene (ribosomal RNA) and *recA* (recombinase A). Three housekeeping genes were tested for *S*. Typhimurium: *gmk* (guanylate kinase), *rpoD* (sigma factor) and 16S gene (ribosomal RNA gene). Because *recA* and *gmk* were the most stable under different treatments, they were selected for normalizing transcript expression levels. The experiments were replicated three independent times. To determine relative changes in gene expression, the formula described by Pfaffl (Pfaffl, 2001) was used: ratio = (E_target_)^ΔCt target(control-sample)^/(E_reference_)^ΔCt reference(control-sample)^, where E is the efficiency of the qPCR, calculated according to the equation: *E* = 10^(−1/slope)^.

**Table 2:** **Primers of virulence genes used for qPCR (F: forward; R: reverse)**.

**Primer**	**Sequence of PCR primers (5′-3′)^a^**	**References**
***E. coli*** **Housekeeping and Virulence Genes**		
*gnd*	F: 5′-GGTAATACCTTCTTCCAGGACACC-3′	Rashid et al., 2006
	R: 5′-TAGTGCGCCCTCCTCACC-3′	
*gst*	F: 5′-CTTTGCCGTTAACCCTAAGGG-3′	Pfaffl, 2001
	R: 5′-GCTGCAATGTGCTCTAACCC-3′	
*recA*	F: 5′-CAATATTCCCCACTGCTGCC-3′	Takle et al., 2007
	R: 5′-CACCTAGGCGACGATCCCT-3′	
*16S*	F: 5′-GGTGAGCTGGTTGATCTGGG-3′	Takle et al., 2007
	R: 5′-GCATTCGCTTTACCCTGACC-3′	
*ler*	F: 5′-TTTCTTCTTCAGTGTCCTTCA-3′	Medellin-Pena et al., 2007
	R: 5′-TGCGGAGATTATTTATTATGA-3′	
*fliC*	F: 5′-TACCATCGCAAAAGCAACTCC-3′	Medellin-Pena et al., 2007
	R: 5′-GTCGGCAACGTTAGTGATACC-3′	
*luxS*	F: 5′-GATCATACCCGGATGGAAG-3′	Medellin-Pena et al., 2007
	R: 5′-AGAATGCTACGCGCAATATC-3′	
*stxB2*	F: 5′-AGATGTTTATGGCGGTTTTA-3′	Medellin-Pena et al., 2007
	R: 5′-TTAAACTGCACTTCAGCAAA-3′	
*qseA*	F: 5′-CGCGGATCCCGTTGGCACAGGTTTGTACA-3′	Medellin-Pena et al., 2007
	R: 5′-CGCGGATCCCGTTGGCACAGGTTTGTACA-3′	
***S***. **Typhimurium Housekeeping and Virulence Genes**		
*gmk*	F: 5′-TTGGCAGGGAGGCGTTT-3′	Rashid et al., 2006
	R: 5′-GCGCGAAGTGCCGTAGTAAT-3′	
*rpoD*	F: 5′-ACATGGGTATTCAGGTAATGGAAGA-3′	Botteldoorn et al., 2006
	R: 5′-CGGTGGGTATTCAGGTAATGGAAGA-3′	
*16S*	F: 5′-AGGCCTTCGGGTTGTAAAGT-3′	Xu et al., 2010
	R: 5′-GTTAGCCGGTGCTTCTTCTG-3′	
*hilA*	F: 5′-TGTCGGAAGATAAAGAGCAT-3′	Guri et al., 2016
	R: 5′-AAGGAAGTATCGCCAATGTA-3′	
*sopD*	F: 5′-ATTAATGCCGGTAACTTTGA-3′	Guri et al., 2016
	R: 5′-CTCTGAAAACGGTGAATAGC-3′	
*ssrB2*	F: 5′-TGGTTTACACAGCATACCAA-3′	Guri et al., 2016
	R: 5′-GGTCAATGTAACGCTTGTTT-3′	

### Statistical analysis

The data are means ± standard error of three replicates. A Student's *t*-test was used to assess the statistical significance of the differences between test and control groups, where *p* ≤ 0.05 was considered as significant.

## Results

### Growth of *Bifidobacterium bifidum* and *Bifidobacterium crudilactis*

*B. crudilactis* FR/62/B/3 showed increase in viable counts in MRS2, MRS2-Wh and MRS2-3′SL compared to MRS2 G, but the highest counts were observed on MRS2-Wh (8.9 ± 0.6 log cfu/ml, Table 3). The same trend was observed for *B. bifidum* BBA1 with slightly lower counts (Table 3) compared to *B. crudilactis* FR/62/B/3. The highest counts were also observed for MRS2-Wh (8.1 ± 0.3 log cfu/ml).

**Table 3:** **Counts of *B. bifidum* and *B. crudilactis* after 48 h of incubation in MRS2 G, MRS2, MRS2-Wh, and MRS2-3′SL media**.

	**Final concentrations after 48 h incubation (log cfu/ml)**	
	***B. bifidum***	***B. crudilactis***
MRS2 G	6.9 ± 0.3	5.5 ± 0.5
MRS2	7.3 ± 0.8	7.8 ± 1.4
MRS2-Wh	8.1 ± 0.3	8.9 ± 0.6
MRS2-3′SL	6.8 ± 0.9	7.9 ± 0.3

### Activity of CFSM from MRS2-3′SL fermented by bifidobacteria on bioluminescent reporter gene expression

**Figure 1:**
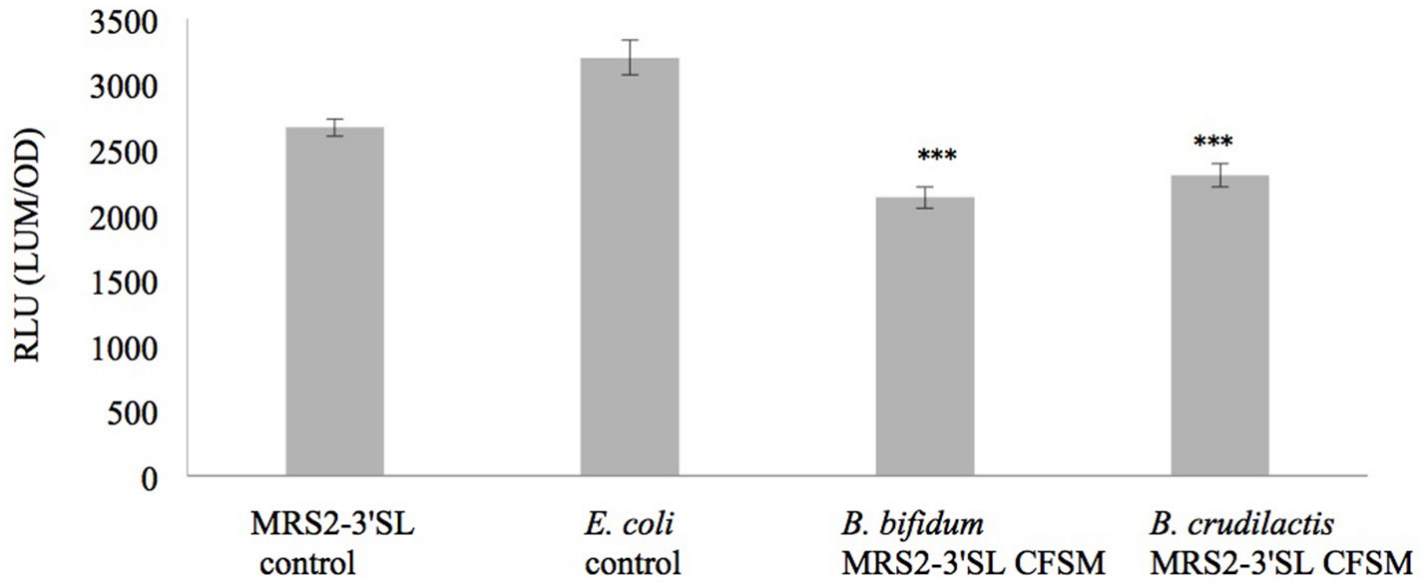
**Effect of CFSM from MRS2-3′SL medium fermented with *B. bifidum* and *B. crudilactis* on *E. coli* O157:H7 (stx^−^, LEE1:*lux*) ATCC 43888 expression**. The *E. coli* control is *E. coli* grown on BHI only. The MRS2-3′SL control is *E. coli* grown on BHI and CFSM from MRS2-3′SL medium unfermented. Data are the means ± the standard deviations derived from triplicate and expressed as relative light units (RLU) defined as counts per seconds, adjusted to OD600 (RLU/OD600) and where OD is fixed at 0.806 and was taken after 4 h of incubation. LUM: luminescence; OD: optical density. ^***^P ≤ 0.005.

**Figure 2:**
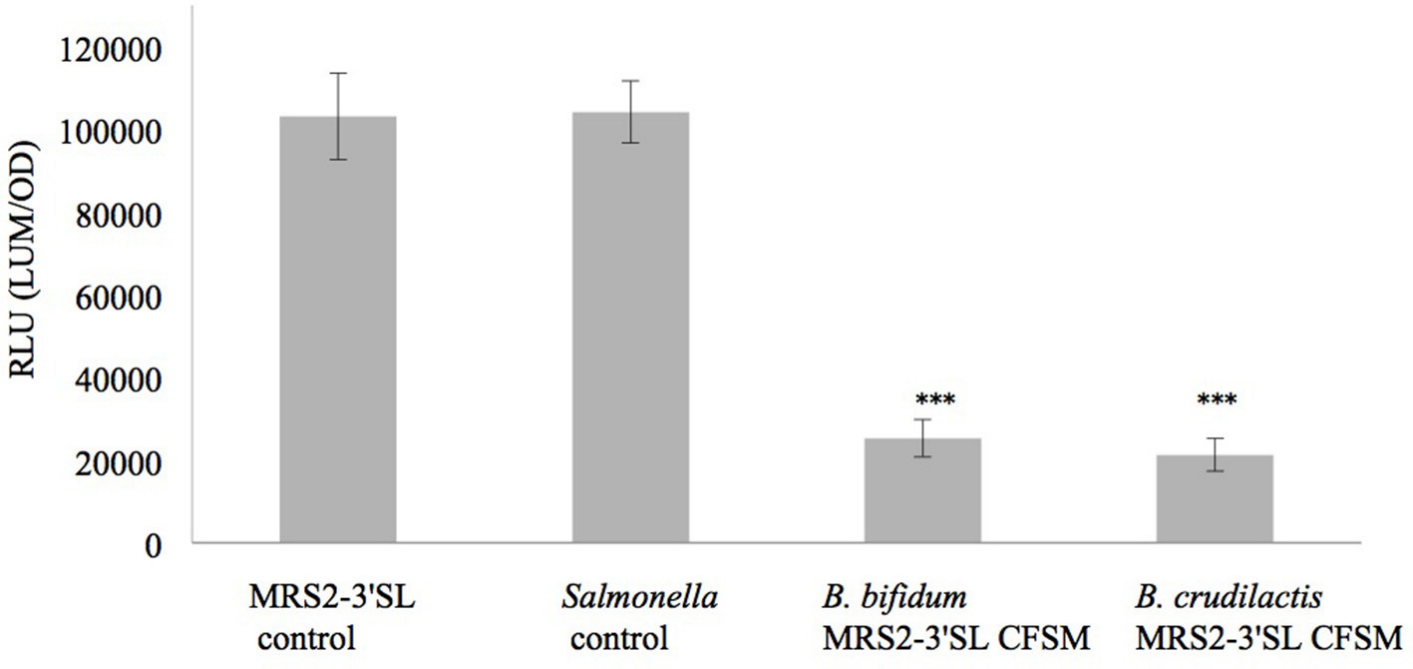
**Effect of CFSM from MRS2-3′SL medium fermented with *B. bifidum* and *B. crudilactis* on *S*. Typhimurium (*hilA*::*lux*) SA 941256 expression**. The *Salmonella* control is *Salmonella* grown on BHI only. The MRS2-3′SL control is *S*. Typhimurium grown on BHI and CFSM from MRS2-3′SL medium unfermented. Data are the means ± the standard deviations derived from triplicate and expressed as relative light units (RLU) defined as counts per seconds, adjusted to OD600 (RLU/OD600) and where OD is fixed at 0.909 and was taken after 13 h of incubation. LUM: luminescence; OD: optical density. ^***^P ≤ 0.005.

Luminescence expression of the plasmids LEE::*lux*CDABE and *hilA:lux*CDABE reached its maximum at 4 h for the *E. coli* mutant and at 13 h for the *S*. Typhimurium mutant, respectively. In the presence of supernatants from fermented MRS2-3′SL medium, bioluminescence induction decreased for both mutants (Figures 1, 2) showing a decrease in promoter expression of *ler* and *hilA*. These results, statistically significant for both strains, were more pronounced for *hilA* gene expression of *S*. Typhimurium (Figure 2).

### Effect of CFSM on *E. coli* O157:H7 virulence gene expression

**Figure 3:**
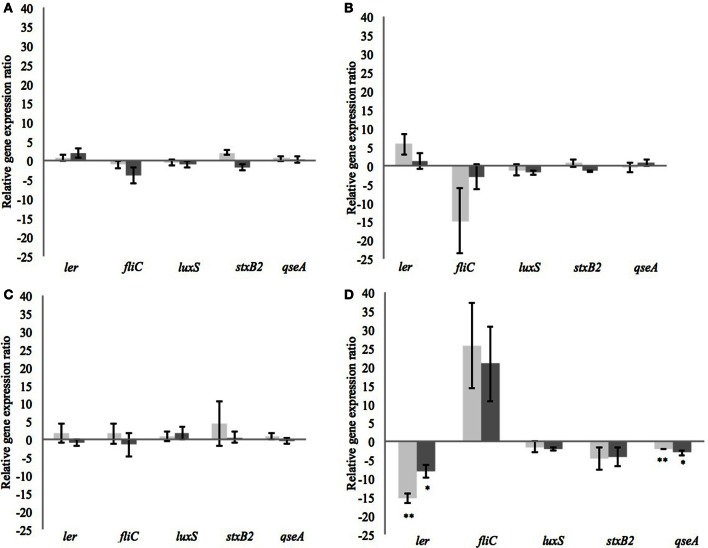
**Effect of CFSM from MRS2 G medium (A), MRS2 medium (B), MRS2-Wh medium (C), and MRS2-3′ SL medium (D) fermented by *B. bifidum* (light gray) and *B. crudilactis* (dark gray) on virulence gene expression of enterohaemorrhagic *E. coli* O157:H7 (EHEC) after 4 h of incubation**. Gene expression ratios of *E. coli* O157:H7 were normalized to the expression of the housekeeping gene *recA* and compared with those of the unfermented media. Negative values represent down-regulation of genes and positive values represent up-regulation of genes. ^*^P ≤ 0.05; ^**^P ≤ 0.01.

After incubation of 4 h, the different CFSM had no negative impact on growth. The average OD of *E. coli* O157:H7 at 600 nm after 4 h exposure was around 1.286 ± 0.119. From every tested housekeeping gene, *recA* was the most stable and was chosen to normalize the results according to the efficiency of each pair of primers (virulence genes *ler, fliC, stxB2, luxS*, and *qseA*) monitored using qPCR (Pfaffl, 2001; Tellez et al., 2012) (Table 4). The fermented MRS2 G, MRS2, and MRS2-Wh media did not show significant impact on gene expression (Figures 3A–C) compared to non-fermented control media, meaning that CFSM had no effects. However, significant down-regulation of virulence genes of *E. coli* O157:H7 was observed in the presence of fermented MRS2-3′SL medium (Figure 3D). The medium fermented by *B. bifidum* BBA1 and *B. crudilactis* FR/62/B/3 induced a down-regulation of the *E. coli ler* gene (ratios of −15.4; *P* ≤ 0.01 and −8.1; *P* ≤ 0.05, respectively). A down-regulation of the *qseA* gene was also observed (ratios of −2.1; *P* ≤ 0.01 and −3.1; *P* ≤ 0.05, respectively). A non-significant trend for up-regulation of the *fliC* gene (ratios of 25.8 and +20.8, respectively) was noted while a non-significant trend for down-regulation of the *stxB2* gene (ratios of −4.6 and −4.2, respectively) was observed. In the case of fermentation by *B. crudilactis* FR/62/B/3, a slight non-significant trend for down-regulation of the *luxS* gene was observed (ratio of −2.1).

**Table 4:** **Effect of CFSM on expression (cycle threshold values ± standard error) of virulence genes of enterohaemorragic *E. coli* O157:H7 after 4 h incubation**.

**Gene**	**PCR efficiency^a^ (%)**	**LB control^b^**	**Test supernatants from unfermented media^c^**				**CFSM from fermented media by *B. bifidum*^d^**				**CFSM from fermented media by *B. crudilactis*^e^**			
			**MRS2 G**	**MRS2**	**MRS2-Wh**	**MRS2-3′SL**	**MRS2 G**	**MRS2**	**MRS2-Wh**	**MRS2-3′SL**	**MRS2 G**	**MRS2**	**MRS2-Wh**	**MRS2-3′SL**
*recA*	100	20	21.3 ± 0.6	18.2 ± 0.5	21.0 ± 0.7	20.3 ± 0.3	21.2 ± 0.4	21.4 ± 1.2	23.6 ± 1.9	19.9 ± 0.9	20.9 ± 0.5	23.7 ± 0.2	21.7 ± 0.3	19.9 ± 1.0
*ler*	93	18.9	25.2 ± 0.4	20.3 ± 1.3	26.4 ± 1.6	19.9 ± 0.8	25.6 ± 1.2	25.4 ± 0.3	28.4 ± 1.6	23.8 ± 0.4	24.2 ± 1.4	25.6 ± 1.0	27.8 ± 2.0	22.8 ± 2.0
*fliC*	97	20.8	18.2 ± 4.0	22.8 ± 1.4	24.1 ± 1.1	23.4 ± 2.0	19.1 ± 0.3	24.9 ± 4.2	26.0 ± 4.9	18.7 ± 1.5	19.8 ± 1.6	21.7 ± 3.7	25.3 ± 3.1	19.3 ± 0.6
*luxS*	91	23.6	26.8 ± 0.9	20.7 ± 1.6	30.1 ± 1.9	25.0 ± 1.3	27.7 ± 1.0	28.3 ± 0.9	32.6 ± 4.7	25.4 ± 1.5	27.4 ± 0.3	26.8 ± 3.1	29.8 ± 3.3	25.8 ± 2.2
*stxB2*	95	26.1	28.8 ± 1.0	25.7 ± 1.0	33.4 ± 2.4	26.5 ± 2.6	29.3 ± 3.0	30.4 ± 1.5	35.3 ± 1.0	27.6 ± 0.5	29.7 ± 2.5	30.1 ± 2.3	33.8 ± 3.8	27.8 ± 2.0
*qseA*	91	22.5	24.1 ± 0.4	26.3 ± 0.4	24.4 ± 0.8	21.9 ± 0.5	24.7 ± 1.4	25.3 ± 2.0	27.0 ± 2.1	22.7 ± 1.2	24.1 ± 0.9	23.5 ± 3.0	25.2 ± 1.0	23.3 ± 1.0

a *PCR efficiency: E = [(10^(-1/slope)^)/2] x 100%*.

b *E. coli O157:H7 grown in LB broth for 4 h*.

c *E. coli O157:H7 grown in LB broth supplemented with unfermented culture media CFSM for 4 h*.

d *E. coli O157:H7 grown in LB broth supplemented with fermented concentrated culture media CFSM from B. bifidum for 4 h*.

e *E. coli O157:H7 grown in LB broth supplemented with fermented concentrated culture media CFSM from B. crudilactis for 4 h*.

### Effect of CFSM on *S*. typhimurium virulence gene expression

**Figure 4:**
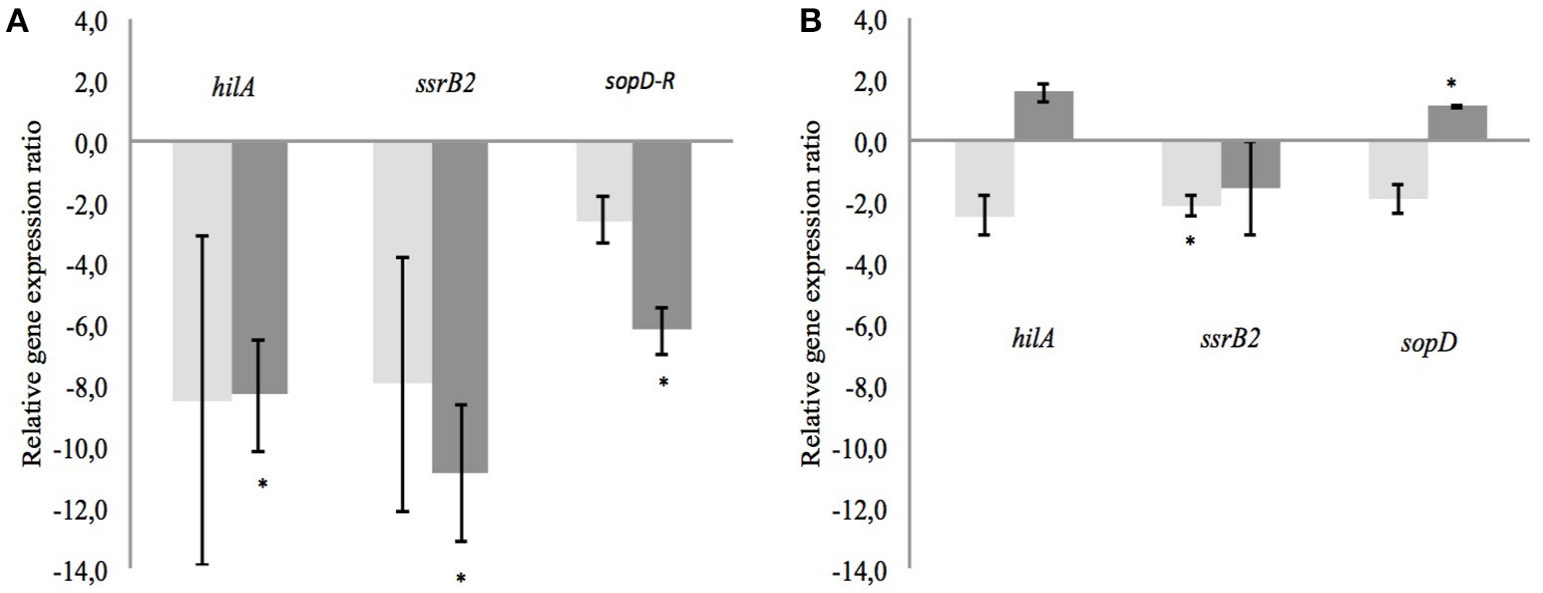
**Effect of CFSM from MRS2 G medium (A) and MRS2-3′ SL medium (B) fermented by *B. bifidum* (light gray) and *B. crudilactis* (dark gray) on virulence gene expression of *S*. Typhimurium after 4 h of incubation**. Gene expression ratios of *S*. Typhimurium were normalized to the expression of the housekeeping gene *gmk* and compared with those of the unfermented media. Negative values represent down-regulation of genes and positive values represent up-regulation of genes. ^*^P ≤ 0.05.

After incubation of 4 h, the OD measurements showed no negative impact on growth. The average OD of *S*. Typhimurium at 600 nm and after 4 h of exposure was 0.862 ± 0.078. From every tested housekeeping gene, *gmk* was the most stable and was chosen to normalize and adjust the results according to the efficiency of each pair of primers (virulence genes *hilA, ssrB2*, and *sopD*) monitored using qPCR (Guri et al., 2016, Table 5). The CFSM of MRS2-3′SL medium fermented by *B. bifidum* induced a slight down-regulation of the *ssrB2* gene (−2.1; *P* ≤ 0.05). The same trend was observed for the genes *hilA* and *sopD* (−2.5 and −1.9, respectively). MRS2-3′SL CFSM fermented by *B. crudilactis* FR/62/B/3 did not show any significant effect on virulence gene expression (Figure 4B). A significant increase of *sopD* expression is observed but too light to be biologically meaningful (1.1; *P* ≤ 0.05), and the same but non-significant trend is observed with *hilA* gene (1.6). Interestingly, a down-regulation of virulence genes was observed with CFSM obtained from MRS2 without glucose. In the case of fermentation by *B. crudilactis* FR/62/B/3, the genes *hilA, ssrB2* and *sopD* were down regulated (−8.3, −10.9, and −6.2, respectively; *P* ≤ 0.05). The same but non-significant trend was observed for *B. bifidum* BBA1 (−8.5, −8.0, and −2.6, respectively, Figure 4A).

**Table 5:** **Effect of CFSM on expression (cycle threshold values ± standard error) of virulence gene expression of *S*. Typhimurium after 4 h incubation**.

**Gene**	**PCR efficiency^a^ (%)**	**BHI control^b^**	**Test supernatants from unfermented media^c^**		**CFSM from fermented media by *B. bifidum*^d^**		**CFSM from fermented media by *B. crudilactis*^e^**	
			**MRS2 G**	**MRS2-3′SL**	**MRS2 G**	**MRS2-3′SL**	**MRS2 G**	**MRS2-3′SL**
*gmk*	100	23	23.3 ± 0.3	24.4 ± 1.2	23.4 ± 0.4	24.4 ± 1.6	21.8 ± 0.2	25.0 ± 1.6
*hilA*	91	31.5	28.6 ± 0.2	31.2 ± 0.7	31.4 ± 2.8	32.5 ± 1.2	30.2 ± 0.4	31.3 ± 2.0
*ssrB2*	115	31.2	28.7 ± 1.1	30.8 ± 0.1	30.9 ± 1.6	31.6 ± 1.6	30.2 ± 0.6	32 ± 2.0
*sopD*	91	30.1	27.7 ± 0.5	30.2 ± 1.0	29.3 ± 1.6	31.2 ± 1.5	28.9 ± 0.6	30.9 ± 2.1

a *PCR efficiency: E = [(10^(-1/slope)^)/2] x 100%*.

b *S. Typhimurium grown in BHI broth for 4 h*.

c *S. Typhimurium grown in BHI broth supplemented with concentrated supernatants from culture media unfermented for 4 h*.

d *S. Typhimurium grown in BHI broth supplemented with concentrated supernatants from culture media fermented B. bifidum for 4 h*.

e *S. Typhimurium grown in BHI broth supplemented with concentrated supernatants from culture media fermented B. crudilactis for 4 h*.

## Discussion

*B. crudilactis* FR/62/B/3 presented the best growth potential compared to *B. bifidum* BBA1, particularly with whey or 3′SL instead of glucose. This could be explained by the fact that *B. crudilactis* FR/62/B/3 was originally isolated from raw cow milk and raw milk cheese. This species possesses the genetic machinery suggesting some ability to synthetize specific enzymes for BMO degradation, as highlighted by the presence of genes coding for β-galactosidase and α- or β-glucosidase, genes also present in other bacteria such as *B. bifidum, B. longum* subsp. *infantis, B. mongoliense, B. biavatii, B. kashiwanohense*, and *B. stellenboschense* (Delcenserie et al., 2007; Milani et al., 2014, 2015; Bondue and Delcenserie, 2015). *B. bifidum* BBA1 grew as well on medium containing 3′SL as the main carbohydrate source. This species possesses genes encoding some of the enzymes cleaving BMO bonds, so their expression would lead to growth. However, the growth results of bifidobacteria on this media were similar to those observed with MRS2 G (control). This could mean that those genes may not be expressed efficiently under the conditions tested, or another function is necessary. A next step would be to measure the expression of genes involved in carbohydrate metabolism such as genes coding for β-galactosidase and α- or β-glucosidase to validate the expression of those genes in the presence of BMO. Another hypothesis could be attributed to the presence of residual glucose from MRS culture allowing bifidobacteria to grow in medium exempt of carbohydrate. Indeed, the glucose present in initial MRS medium was in excess (Tanimomo et al., 2016), but a negligible part of it (maximum 1 mg) has been transferred with the inoculum. Another source of glucose could be the presence of residual carbohydrate in the yeast extracts or peptone extracts used in MRS media. The positive effects of media supplemented with milk products on growth of probiotics has been demonstrated previously (Champagne et al., 2014). This is confirmed in the present study as the best levels of growth were reached on MRS2-Wh media for both strains studied. In addition to BMO, whey is rich in lactose (Food and Agriculture Organization/Organisation Mondiale de la Santé, 1998), a carbohydrate source that is easily consumed by bifidobacteria (Delcenserie et al., 2007).

As demonstrated previously, the CFSM obtained from some lactic acid bacteria or bifidobacteria can induce a decrease in virulence gene expression of pathogenic bacteria such as *C. jejuni* (Mundi et al., 2013). Likewise, *B. bifidum* ATCC 29521, and *L. acidophilus* La-5 CFSM were able to produce metabolites inhibiting virulence gene expression of *E. coli* O157:H7 (Medellin-Pena et al., 2007) and *S*. Typhimurium (Bayoumi and Griffiths, 2012). In our study, CFSM collected from MRS2-3′SL medium fermented by *B. bifidum* BBA1 and *B. crudilactis* FR/62/B/3 down-regulated most of the virulence genes tested in *E. coli* O157:H7, except the *fliC* gene, which tended to be up-regulated. This is not surprising according to the fact that *fliC* gene is not coded by the LEE operon and therefore not necessarily regulated as other virulence genes involved in T3SS and situated within the LEE operon (Falcao et al., 2004). No significant effect has been observed with CFSM from MRS2 and MRS2-Wh medium. In addition, CFSM obtained from bifidobacteria grown in media enriched in 3′SL were able to affect virulence gene expression of *E. coli* O157:H7 without having any impact on its growth, at least during the first 4 h of incubation.

In those media, higher in carbohydrates, more fermentation products such as lactate or acetate are synthetized and could have an inhibiting effect on pathogenic bacteria, as well as acidifying the media. However, all CFSM were neutralized before testing them against *E. coli* or *Salmonella*, meaning that the pH did not exert any effect on *E. coli* O157:H7 growth. Furthermore, under neutral pH, the organic acids were under dissociated form and should not present any bactericidal or bacteriostatic action, contrary to un-dissociated forms (Momose and Hirayama, 2008).

The genes involved in virulence expression such as *ler* but also *fliC* genes are regulated by *luxS*, involved in quorum sensing. However, nutrients can interfere with quorum sensing mechanisms (Henke and Bassler, 2004; Kaper et al., 2004; Nakanishi et al., 2006; Mellies et al., 2007) and induce a decrease in virulence gene expression through a decrease in *luxS* expression. Delcenserie et al. (2012) previously demonstrated the effects of glucose in down-regulating virulence gene expression of *E. coli* O157:H7. The present study brought out similar observations with lactose instead of glucose (data not shown). The *ler* gene was the most affected by the presence of those carbohydrates and the effect was dose-dependent. Media used as controls and containing glucose or lactose (MRS2 and MRS2-Wh) down-regulated this gene but no effect was observed with medium containing mainly 3′SL as a source of carbohydrate (MRS2-3′SL).

To be able to metabolize 3′SL, *B. bifidum* and *B. crudilactis* have to secrete sialidases through which NeuAc (sialic acid) can be produced. *B. bifidum* does not use this sialic acid, which is available for other bacteria such as *B. breve* (Milani et al., 2015). Therefore, if 3′SL is metabolized by *B. bifidum*, free sialic acid was probably present in fermented CFSM from 3′SL medium. Usually, pathogenic bacteria are able to bind this free sialic acid to their cell surface and use it to improve their resistance to the host's innate immune response, or can consume it as a nutrient (Vimr et al., 2004; Severi et al., 2007). NeuAc also exerts a major role in *Salmonella enterica* subsp. *enterica* serovar Typhi adhesion to intestinal epithelium (Sakarya et al., 2010). This means that in theory, sialic acid could have an impact on *S*. Typhimurium and *E. coli* growth, but no effect on growth as measured by OD has been observed. In addition, NeuAc had probably no impact on virulence gene expression in our study, when supplied in the medium.

The non-significant trend for up-regulation of *fliC* observed in our study should be clarified. Indeed, this trend seems higher when *E. coli* O157:H7 was exposed to CFSM from fermented 3′SL. A hypothesis could be that the presence of residual complex carbohydrates affects gene expression of *fliC*. The presence or absence of some nutrients, or stress, could play a role in virulence expression, including *fliC* (Mei et al., 2015). Several studies investigated the effects of some stress (oxidative stress, heat shock, long storage) on down-regulation of *fliC* gene while other virulence genes were upregulated (Carey et al., 2009; Mei et al., 2015; Singh and Jiang, 2015). The experimental protocol of this study submitted *E. coli* O157:H7 to some stress, which may influence virulence gene expression. Genes involved in general stress (*uspA* and *rpoS*), in starvation (*phoA* and *dpS*), in cold shock (*cspA, cspC*, and *cspE*) and in acid resistance (*gadW*) have been investigated and the results have shown that the different treatments did not affect expression of stress-related genes (data not shown). This leads us to suppose that virulence factor expression has not been influenced by the experimental conditions.

The results observed using RT-qPCR with CFSM from fermented MRS2-3′SL on *S*. Typhimurium virulence gene expression did not confirm the results observed with luminescent reporter strains. However, CFSM obtained after fermentation of MRS2 without any glucose fermented by *B. crudilactis* FR/62/B/3 down-regulated several virulence genes. Regarding *B. bifidum* BBA1 CFSM, a trend to down-regulation was observed as well. These down-regulations could be caused by other non-carbohydrate metabolites produced by bifidobacteria and these bioactive molecules could originate from the degradation of proteins. Most known bioactive molecules, such as subpeptin JM4-A and subpeptin JM4-B are antimicrobial peptides synthetized by *Bacillus subtilis* and active against *Salmonella, Staphylococcus aureus*, and *Bacillus cereus* (Sumi et al., 2015). Nisin, a bacteriocin well known, is produced by *Lactococcus lactis* and has a negative impact on *Listeria* or *Clostridium* (Ebbensgaard et al., 2015). The results of our study suggest that the CFSM activity is not due to an antimicrobial effect but due to an antivirulent effect.

In conclusion, this study provides the information that CFSM obtained from MRS2-3′SL medium fermented by *B. bifidum* BBA1 and *B. crudilactis* FR/62/B/3 down-regulated LEE1 expression of the luminescent *E. coli* reporter strain and *hilA* expression of luminescent *S*. Typhimurium reporter strain. These results agree with the decreasing virulence gene expression of *ler* and *qseA* for *E. coli*, but not for *S*. Typhimurium. The contact between *S*. Typhimurium and CFSM from fermented MRS without glucose showed down-regulation of genes *hilA, ssrB2*, and *sopD*. According to this *in vitro* study, the antivirulent metabolites issuing from fermentation by bifidobacteria could have a negative impact on T3SS of both pathogens, decreasing expression of genes mainly implicated in this virulence mechanism (*ler* and *qseA* genes for *E. coli* O157:H7; *hilA, ssrB2*, and *sopD* genes for *S*. Typhimurium). The potential upregulation of *fliC* in *E. coli* O157:H7 could increase the motility as well as biofilm formation. A phenotypic analysis of the pathogens under the experimental conditions could bring more insights about its virulence pattern. Information is lacking about the nature of the active molecules, but the activity of those CFSM might be due to small peptides or proteins with low molecular weight and resistant to pH modification and heat, or products obtained from carbohydrate metabolism. Size exclusion chromatography could contribute to separating and isolating these bioactive molecules in order to identify them. In the future, the effects of these metabolites will be investigated in a human gastrointestinal model to study the impact on microbiota to mimic *in vivo* conditions.

## Author contributions

PB did the experiments, interpreted the results and wrote the manuscript. SC and FB participated to the experiments. MS, GD, GL, and MG were involved in the design of the study and provided help for interpretation of the results. VD participated to the design of the study, interpretation of the results and writing of the manuscript.

### Conflict of interest statement

The authors declare that the research was conducted in the absence of any commercial or financial relationships that could be construed as a potential conflict of interest.

## Abbreviations

3′SL: 3′-sialyllactose
AE: attaching and effacing
BHI: brain heart infusion
BMO: bovine milk oligosaccharide
CFSM: cell-free spent medium
EHEC: enterohaemorragic *Escherichia coli*
FOS: fructo-oligosaccharide
Fuc: fucose
Gal: galactose
Glc: glucose
GlcNAc: N-acetylglucosamine
GOS: galacto-oligosaccharide
HMO: human milk oligosaccharide
LacNac: N-acetyllactosamine
LB: Luria Bertani
LNB: lacto-N-biose
LNnT: lacto-N-neotetraose
LNT: lacto-N-tetraose
MRS: De Man, Rogosa and Sharpe medium
NeuAc: N-acetylneuraminic acid or sialic acid
NeuGc: N-glycolylneuraminic acid
OD: optical density
T3SS: type III secretion system.
