# Corrigendum: Cell-Free Spent Media Obtained From *Bifidobacterium bifidum* and *Bifidobacterium crudilactis* Grown in Media Supplemented with 3′-Sialyllactose Modulate Virulence Gene Expression in *Escherichia coli* O157:H7 and *Salmonella* Typhimurium

**DOI:** 10.3389/fmicb.2019.02490

**Published:** 2019-11-14

**Authors:** Pauline Bondue, Sébastien Crèvecoeur, François Brose, Georges Daube, Marie-Christine Seghaye, Mansel W. Griffiths, Gisèle LaPointe, Véronique Delcenserie

**Affiliations:** ^1^Department of Food Science, Fundamental and Applied Research for Animal and Health, Faculty of Veterinary Medicine, University of Liège, Liège, Belgium; ^2^Department of Pediatrics, University Hospital Liège-Notre-Dame des Bruyères, Belgium; ^3^Canadian Research Institute for Food Safety, University of Guelph, Guelph, Canada

**Keywords:** *Bifidobacterium bifidum*, *Bifidobacterium crudilactis*, bovine milk oligosaccharide, *Escherichia coli* enterohemorragic O157:H7, *Salmonella enterica* serovar Typhimurium, virulence expression, 3′-sialyllactose, whey

In the original article, there was an error. “The Escherichia coli O157:H7 ATCC 43890 strain mentioned in the original manuscript is not the one used, since it was the Escherichia coli O157:H7 ATCC 35150 strain.”

A correction has been made to the section **MATERIALS AND METHODS**, subsection **Bacterial Strains and Growth Conditions**:

*Bifidobacterium bifidum* BBA1 was isolated from feces from a breast-fed child (CHU - Hôpital des Bruyères, Liège, Belgium) and *B. crudilactis* FR/62/B/3 from Saint-Marcellin, a raw cow milk cheese from Vercors (France). Both strains were stored at −80°C and grown on De Man, Rogosa, and Sharpe (MRS) medium (Oxoid, Hampshire, UK) supplemented with cysteine-HCl (0.5 g/l) and mupirocin (0.08 g/l) at 37°C for 48 h in an anaerobic workstation (Led Techno, Heusden-Zolder, Belgium) containing 10% H_2_, 10% CO_2_, and 80% N_2_. Several successive cultures, in the same conditions as described previously, have been realized in MRS broth, prior to use. Pathogenic enterohaemorrhagic *E. coli* (EHEC) strain O157:H7 ATCC 35150 (${stx}_{2}^{+}$) and *S. enterica* serovar Typhimurium strain ATCC 14028 were stored at −80°C and grown in Luria Bertani (LB) media (Sigma-Aldrich, Diegem, Belgium). Two reporter mutants, *E. coli* O157:H7 ATCC 43888 (*stx*^−^, LEE:*lux*) containing plasmid LEE1-luxCDABE and resistant to ampicillin (Amp^r^) and kanamycin (Kan^r^) and *S*. Typhimurium SA 941 256 containing plasmid pSB377 (*hil*A::*lux*CDABE; Amp^r^) were designed by Medellin-Pena et al. (2007) and Bayoumi and Griffiths (2010), respectively. Both strains were from the Canadian Research Institute for Food Safety Collection and were grown under aerobic conditions at 37°C in brain heart infusion (BHI) broth (Bio-Rad, Marnes-la-coquette, France) supplemented with ampicillin (50 mg/l). A medium optimized for *B. crudilactis* FR/62/B/3, called MRS2 (Tanimomo et al., 2016) was considered as the reference medium for this study (Table 1) and was modified by removing or replacing glucose: MRS2 without any glucose (MRS2 G) (control), MRS2 with a mix of glucose and whey (MRS2-Wh) and MRS2 with 3′SL (MRS2-3′SL) as the only source of carbohydrate (Table 1). Whey was collected at the beginning of a curdling process of a Belgian cheese factory (Liège area, Belgium). The quantity of lactose in MRS2-Wh medium was estimated to 25 g/l, based on lactose concentration of sweet whey (50 g/l of lactose; Food and Agriculture Organization/Organisation Mondiale de la Santé, 1998). However, mature bovine milk contains only traces of BMO (Kelly et al., 2013). The 3′SL, added to MRS2-3′SL, was provided by Carbosynth laboratory (Berkshire, UK). The concentration of 0.85 g/l was chosen to be close to natural concentrations found in colostrum (Nakamura et al., 2003). *B. bifidum* BBA1 and *B. crudilactis* FR/62/B/3 were grown in three independent experiments under the same anaerobic conditions as previously at 37°C for 48 h. Five log/ml of bifidobacteria from a fresh 48 h culture of bifidobacteria were inoculated into the fresh media (1% v/v). The concentration of 5 log/ml was confirmed by plating several dilutions of bifidobacteria at day 0 post inoculation. Bacterial growth was determined by viable counts after 48 h incubation. Cell free spent media (CFSM) were obtained after two centrifugation steps at 5000 × (Eppendorf Centrifuge 5804, Hamburg, Germany) for 10 min. Supernatants were then sterilized by filtration (Minisart® 0.45 μm and 0.2 μm, Sartorius, Vilvoorde, Belgium). Next, CFSM were freeze-dried (Virtis Benchtop 3.3 EL, SP Scientific, Suffolk, United-Kingdom) and rehydrated with sterile distilled water to obtain a 10x concentration. The same treatment was applied to non-fermented culture media (controls). The pH of rehydrated CFSM was adjusted to 7 using 1 M NaOH.

The authors apologize for this error and state that this does not change the scientific conclusions of the article in any way. The original article has been updated.

## References

[B1] Bayoumi M. A. Griffiths M. W. (2010). Probiotics down-regulate genes in *Salmonelle enterica* serovar Typhimurium pathogenicity islands 1 and 2. J. Food Prot. 73, 452–460. 20202329 10.4315/0362-028x-73.3.452

[B2] Food Agriculture Organization/Organisation Mondiale de la Santé (FAO/OMS) (1998). Le lait et les Produits Laitiers dans la Nutrition Humaine. Available online at: http://www.fao.org/docrep/t4280f/t4280f0h.htm (accessed June 16, 2016).

[B3] Kelly V. Davis S. Berry S. Melis J. Spelman R. Snell R. (2013). Rapid, quantitative analysis of 3′- and 6′-sialyllactose in milk by flow-injection analysis-mass spectrometry: screening of milks for naturally elevated sialyllactose concentration. J. Dairy Sci. 96, 7684–7691. 10.3168/jds.2013-6972 24140337

[B4] Medellin-Pena M. J. Wang H. Johnson R. Anand S. Griffiths M. W. (2007). Probiotics affect virulence-related gene expression in *Escherichia coli* O157:H7. Appl. Environ. Microbiol. 73, 4259–4267. 10.1128/AEM.00159-07 17496132 PMC1932779

[B5] Nakamura T. Kawase H. Kimura K. Watanabe Y. Ohtani M. Arai I. (2003). Concentrations of sialyloligosaccharides in bovine colostrum and milk during the prepartum and early lactation. J. Dairy Sci. 86, 1315–1320. 10.3168/jds.S0022-0302(03)73715-1 12741556

[B6] Tanimomo J. Delcenserie V. Taminiau B. Daube G. Saint-Hubert C. Durieux A. (2016). Growth and freeze-drying optimization of *Bifidobacterium crudilactis*. Food Nutr. Sci. 7, 616–626. 10.4236/fns.2016.77063

